# Clinical signs, morphological and phylogenetic characterization of *Myxozoan spp.* infecting Nile tilapia, *Oreochromis niloticus* and African catfish, *Clarias gariepinus* in Qalyubia Governorate, Egypt

**DOI:** 10.1186/s12917-024-04378-0

**Published:** 2024-11-27

**Authors:** Doaa A. Yassen, Eman A. Abd El-Gawad, Khaled A. Abd El-Razik, Karima F. Mahrous, Amany A. Abbass

**Affiliations:** 1 Aquatic Animal Medicine Department, Faculty of Veterinary Medicine, Benha University, Moshtohor, Toukh, Qalyubia 13736 Egypt; 2 Animal Reproduction Department, Veterinary Research Institute, National Research Centre (NRC), Dokki, Cairo 12622 Egypt; 3 Cell Biology Department, Biotechnology Research Institute, National Research Centre (NRC), Dokki, Cairo 12622 Egypt

**Keywords:** African catfish, *Henneguya suprabranchiae*, *Myxobolus brachysporus*, *Myxobolus tilapiae*, Phylogenetic analysis, Nile tilapia

## Abstract

**Context:**

Myxosporean endoparasites (phylum cnidarian) are critical pathogens that affect both wild and cultured freshwater and marine water fishes globally causing huge economic losses and high mortalities.

**Study objective:**

The present study investigated myxosporean infections in Nile tilapia and African catfish collected from the natural resources.

**Methods:**

A total of four hundred Nile tilapia with an average weight (60 ± 5 g) and two hundred African catfish with an average weight (185 ± 30 g) were collected seasonally from Qalyubia Governorate, Egypt for parasitological and molecular diagnosis of isolated myxozoan species.

**Results:**

Microscopic examination revealed *Myxobolus heterosporous*, *Myxobolus brachysporus*, *Myxobolus tilapiae*, and *Myxobolus amieti* in Nile tilapia and *Henneguya suprabranchiae*, and *Myxobolus brachysporus* in African catfish. Sequencing of 18S rDNA gene for isolated *Myxozoan spp*. from Nile tilapia revealed *Myxobolus tilapiae* deposited in GenBank under accession numbers (OR766325 and OR766326). In African catfish, the isolated *Myxobolus brachysporus* sequence was deposited under accession numbers (OR766327 and OR766328). *Henneguya suprabranchiae* was also identified in African catfish (accession. No. OR763724 and OR763433**).**

**Conclusion:**

Overall, these results indicate a high prevalence of myxozoan infection in naturally inhabiting Nile tilapia and African catfish. Curiously, *Henneguya suprabranchiae* was detected in the digestive tract and kidneys of African catfish, which is considered a rare form.

**Implication:**

This study highlighted the importance of parasitic surveys in natural resources that impact fish production.

**Supplementary Information:**

The online version contains supplementary material available at 10.1186/s12917-024-04378-0.

## Introduction

Myxozoans are the most significant microscopic obligate cnidarian parasites that cause severe diseases in wild and cultured fishes all over the world [1, 2]. The complex myxozoans life cycle includes annelids and bryozoans as final invertebrate hosts and fishes as intermediate vertebrate hosts [3, 4]. Transmission is accomplished by two definite types of waterborne spores: actinospores in invertebrate hosts and myxospores in vertebrate hosts [5]. Myxozoans are a highly diverse group of parasites consisting of 2554 species [6] and their identification is mainly based on spore morphology [7, 8]. In addition, molecular characterization has been developed as a crucial tool to diagnose a wide range of new myxozoan species in combination with spore morphology [9, 10] and to ascertain its phylogenetic position within metazoans [11].

In Egypt, different myxozoan species have been recorded from different organs of wild and cultured Nile tilapia, such as Myxospora *sp.* [12], *M. agolus*, *M. heterosporus* type 2, *M. clarri*, *M. heterosporous* (type 3) [13, 14], *Myxobolus dermatobius* [15], *Myxobolus cerebralis* [16], *Myxobolus brachysporus* [17, 18], and *Myxobolus tilapiae* [19, 20].

*Henneguya Thèlohan*, 1892 is one of the most important genera of the subphylum Myxozoa and contains more than 200 described species [21]. This genus is distinguished from other members of myxobolidae based on the morphology of its symmetrical spores along with the presence of paired polar capsules and two caudal projections [22]. Although the presence of two caudal projections is an important characteristic of this genus, the previous molecular studies indicate that *Henneguya* is polyphyletic and that the character has independently arisen several times in *Myxobolidae* [23, 24]. *Henneguya suprabranchiae*, has been found to cause economic losses in catfish farms [25–28]. In addition, *H. exilis* causes serious mortality in mature catfish [29] and *Henneguya ictaluri* causes a proliferative gill disease [30]. Microscopic examination of *Henneguya* infection is the most common step in diagnosis as its spores appear elongated with a two-rounded polar capsule and long tail (spermatozoa like) [31].

Nile tilapia is the main fish species inhabiting the Nile River and one of the least expensive and most readily available fish for people [32]. It is also the most cultured freshwater species due to its many benefits including the fact that it survives in low environmental conditions, disease resistance, fast growth, and high meat quality [33]. African catfish, *Clarias gariepinus*, are also a popular species in the Nile River and its tributaries and have been cultured with other fish species to enhance productivity [31, 33]. In Egypt, the capture fish production from the Nile River, lakes, and sea (Red Sea and Mediterranean Sea) accounts for 21.27% of total Egyptian production in 2021. The largest percentage of caught fish arose from the northern lakes [33] which represents 12.77% of the total catch [34]. Meanwhile, production in the Nile River decreased to 3.72% compared to 3.96% in 2020 [34]. Decreases in fish production from natural resources may be due to several factors including environmental pollution and global warming which affect fish immunity and increase their susceptibility to infectious diseases. In addition, rising water temperatures and high carbon dioxide represent a critical threat to ecosystems because they accelerate the parasite life cycle and are consequently able to multiply rapidly in aquatic environments [35]. Disease outbreaks in fish are the most devastating challenge for fish production. Many freshwater fish species are seriously afflicted with various parasites, resulting in high fish mortality, reduced productivity, and negative influence on the economy [2, 36]. Nearly 80% of disease outbreaks in Egypt are due to parasitic infections [37]. Therefore, the current study was designed to investigate the prevalence of *Myxozoan* species infecting Nile tilapia and African catfish naturally from the El-Riah El-Tawfiki canal in Toukh, Qalyubia Governorate, lower Egypt.

## Materials and methods

### Samples collection

**Fig. 1:**
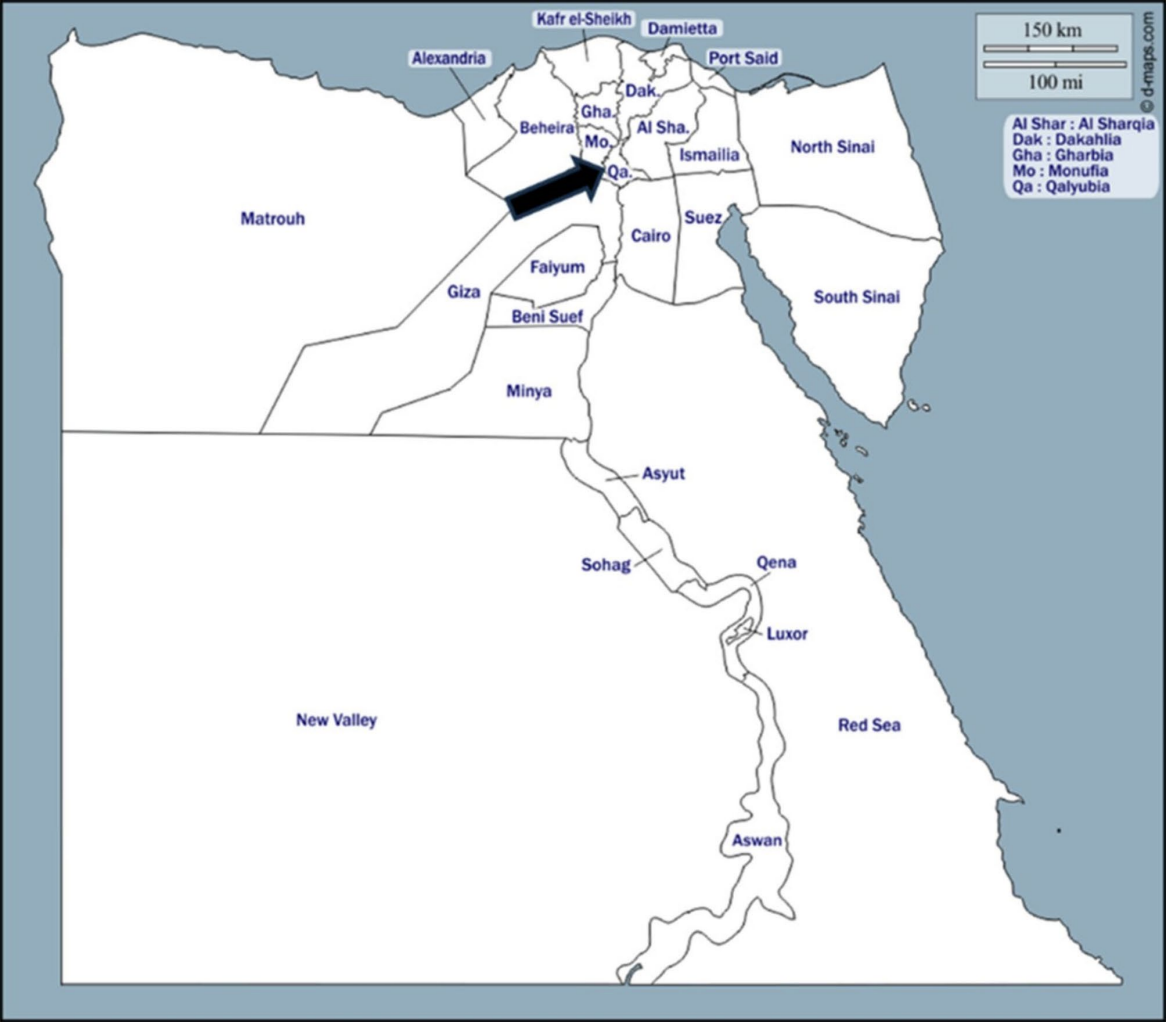
A map showing the location of studying area in Toukh, Qalyubia Governorate, Egypt

A total of four hundred Nile tilapia weighing an average of (55–65 g) and two hundred African catfish weighing an average of (150–210 g) were examined between December 2020 and September 2021. Fish were purchased from fishermen who caught them naturally from the El-Riah El-Tawfiki streams, in Toukh, Qalyubia Governorate, Egypt (Fig. 1). All fish samples were immediately transported to the Diagnostic Laboratory of Aquatic Animal Medicine Department, Faculty of Veterinary Medicine, Benha University, Egypt for clinical and parasitological examinations.

### Clinical and parasitological examination

Fish were subjected to external examination of the body surface, euthanized by an overdose of MS-222 (Sigma) at a dose of 150 mg/L, and sacrificed for internal examination according to Austin et al. [38]. Wet mount slides were prepared from naturally infected fishes showing white nodules or cysts externally or internally. These slides were prepared by gently opening the nodules using a fine needle on a clean glass slide, and the milky fluid was mixed with a drop of distilled water for microscopic examination. The positive slides were air-dried, fixed with absolute methyl alcohol (Sigma-Aldrich) and stained with Giemsa according to the method described by Abdel Ghaffar et al. [17]. The photographed slides were imported to Image J software for morphometric dimensions of spores and polar capsules of each observed spore according to Lom and Arthur [39] criteria.

### Molecular identification and phylogenetic analysis

The Genomic DNA from the recovered *Myxozoan spp.* was extracted following manufacturer’s instructions for GF-1^®^ Tissue DNA extraction Kit (Vivantis, Malaysia). A Nano Drop™ ND-1000 Spectrophotometer (Thermo Scientific, Germany) was used to measure the quality and concentration of the extracted DNA. The extracted DNA was stored at -20 ^◦^C for further molecular analysis.

A fragment of 18S ribosomal DNA (18S rDNA) genes from the retrieved *Myxo*zoan *spp.* was amplified using the universal primer pair 18e and 18 g and universal primers, MX5 and MX3 for *Henneguya spp.* as described by Andree et al. [40]. The amplification assay was performed as described by Eszterbauer [41] and Abdel-Gaber et al. [42]. The second nested PCR amplification was performed using the primer pair MX5 and MX3 [40] for *Myxozoan spp.* and the myxozoan-specific primers; MC5 and MC3 [43] for *Henneguya spp.* (Table 1).

**Table 1:** List of PCR primers used in the present study with amplicons size and references

Primer	Sequence (5´-3´)	Amplicons size (bp)	Reference
18e	CTGGTTGATTCTGCCAGT	1600 bp	Andree et al. [40]
18G	CGGTACTAGCGACGGGCGGTGTG		
MX5	CTGCGGACG GCTCAGTAAATCAGT	1300 bp	Andree et al. [40]
MX3	CCAGGACATCTTAGGGCATCACAGA		
MC5	CCTGAGAAACGGCTACCACATCCA	1000 bp	Molnár et al. [43]
MC3	GATTAGCCTGACAGATCACTCCACGA		

Polymerase Chain Reaction (PCR) was conducted in a final volume of 25 µl reaction mixture comprising 12.5 µl of 2x MyTaq™ Red Mix (Cat. BIO-25043, Meridian Life Science Inc., USA), 0.5 µl of each primer (10 Mmol), and (2 µl) of aim DNA. The PCR conditions were as follows: initial denaturation for 4 min at 94 ◦C, followed by 40 cycles of 94 ◦C for 30s, 56 ◦C for 60s, and 72 ◦C for 90 s, with a final extension at 72 ◦C for 7 min. PCR products were subjected to electrophoresis in 1.5% agarose gel in a TAE buffer, stained with ethidium bromide stain (Merck, Germany), and then analyzed with a gel documentation system. For sizing and approximate quantification of double-stranded DNA, a 1 kb plus DNA Ladder (Vivantis, Malaysia) was used. The Gene JET Gel Extraction Kit (K0691, Thermo Fisher, USA) was used to clean two of the positive PCR products targeting 18S rDNA genes from *Myxozoan spp.* in Nile tilapia and African catfish. The sequences were then run by Macrogen Company (Korea). Two-way sequencing using the specific primers used in PCR confirmed the accuracy of the data. The programs Bioedit 7.0.4.1 and MUSCLE were used to examine the nucleotide sequences acquired in this work. Using a neighbor-joining technique for the aligned sequences implemented in the application CLC 6, the obtained sequences were aligned with reference sequences genes of *Myxozoan species* (Table 2).

**Table 2:** 18S rDNA gene sequences of *Myxozoan spp.* from GenBank used for phylogenetic tree construction

Access No	Species	Host	Isolation Source	Country
OR766325 (our study)	*Myxobolus tilapiae*	*Oreochromis niloticus*	Kidneys	Egypt
OR766326 (our study)	*Myxobolus tilapiae*	*Oreochromis niloticus*	Kidneys	Egypt
OR766327 (our study)	*Myxobolus brachysporus*	*Clarias gariepinus*	Kidneys	Egypt
OR766328 (our study)	*Myxobolus brachysporus*	*Clarias gariepinus*)	Kidneys	Egypt
OR763724 (our study)	*Henneguya suprabranchiae*	*Clarias gariepinus*	Kidneys	Egypt
OR763433 (our study)	*Henneguya suprabranchiae*	*Clarias gariepinus*	Dentritic organs	Egypt
KX632950	*Myxobolus tilapiae*	*Oreochromis niloticus*	Kidneys	Egypt
MZ090095	*Myxobolus tilapiae*	*Oreochromis niloticus*	……………….	Egypt
KX632947	*Myxobolus fomenai*	*Oreochromis niloticus*	Kidneys	Egypt
KX632949	*Myxobolus brachysporus*	*Oreochromis niloticus*	Kidneys	Egypt
KX632948	*Myxobolus agolus*	*Oreochromis niloticus*	Kidneys	-------
KX632951	*Triangula egyptica*	*Oreochromis niloticus*	Kidney	Egypt
AY479924	*Myxobolus cerebralis*	*actinospores*	………….	USA
KP990670	*Myxobolus sp.*	*Oreochromis niloticus*	Spleen	Egypt
KU297653	*Myxobolus sp.*	*Oreochromis niloticus*	Eye	Egypt
JN003830	*Myxobolus arcticus*	*Lumbriculus variegatus*	……….	Japan
FJ417067	*Myxobolus lentisuturalis*	*Carassius gibelio*	Muscles	China
JQ690363	*Myxobolus ampullicapsulatus*	*Carassius auratus auratus*	Gills	China
FJ981819	*Myxobolus notropis*	*Notropis atherinoides*	--------	USA
JQ690360	*Myxobolus nielii*	*Carassius auratus auratus*	Gills	China
FJ417053	*Henneguya doneci*	*Carassius auratus auratus*	Gill filament	China
JQ690372	*Thelohanellus wuhanensis*	*Carassius auratus gibelio*	Skin	China
L39110	*Ichthyosporidium sp.*	------------	---------	-------
MT231728	*Henneguya sp.* Qena1	*Clarias gariepinus*	Gills	Egypt
MT231729	*Henneguya sp.* Qena2	*Clarias gariepinus*	Stomach	Egypt
MT231730	*Henneguya sp.* Qena3	*Clarias gariepinus*	Intestine	Egypt
MN497413	*Myxobolus sp. n. LM-2019*	*Opsaridium ubangiense*	Skin, muscles and spleen	Cameroon
MN733742	*Henneguya fusiformis*	*Clarias gariepinus*	ovaries	Egypt
AF021881	*Henneguya exilis*	*Ictalurus punctatus*	Gills	USA
AF195510	*Henneguya ictaluri*	*Ictalurus punctatus*	Gills	USA
EU492929	*Henneguya adiposa*	*Ictalurus punctatus*	Adipose fin	USA
KP404438	*Henneguya mississippiensis*	*Ictalurus punctatus*	gills	USA
EF191200	*Henneguya sutherlandi*	*Ictalurus punctatus*	Epidermal plasmoidia	USA
JN201199	*Henneguya suprabranchiae*	*Clarias gariepinus*	Gill filaments	Egypt
KX354352	*Henneguya laseeae*	*Pylodictis olivaris*	Gill tissue	USA
FJ468488	*Henneguya pellis*	*Ictalurus furcatus*	external/internal pseudocyst	USA
AF306794	*Henneguya lesteri*	*Sillago analis*	gill and pseudobranch	Australia
GQ340975	*Henneguya tunisiensis*	*Symphodus tinca*	Gill arch	Tunisia
AB183747	*Henneguya lateolabracis*	*Lateolabrax sp.*	heart tissues	Japan
AB183748	*Henneguya pagri*	*Pagrus major*	heart tissues	Japan
U37549	*Henneguya doori*	*Perca flavescens*	Gill filaments	Canada
AF031411	*Henneguya salminicola*	*Oncorhynchus nerka*	………………….	British Columbia, Canada
KT072742	*Unicauda fimbrethilae*	*Ictalurus punctatus*	Intestinal tract	USA

### Statistical analysis

The seasonal and total prevalence of myxozoan infection among the examined fishes were assessed based on the obtained data using the following formula [44]; prevalence (%) = (∑ infected fish/ ∑ fish examined × 100).

## Results

### Clinical signs of naturally infected fishes

**Fig. 2:**
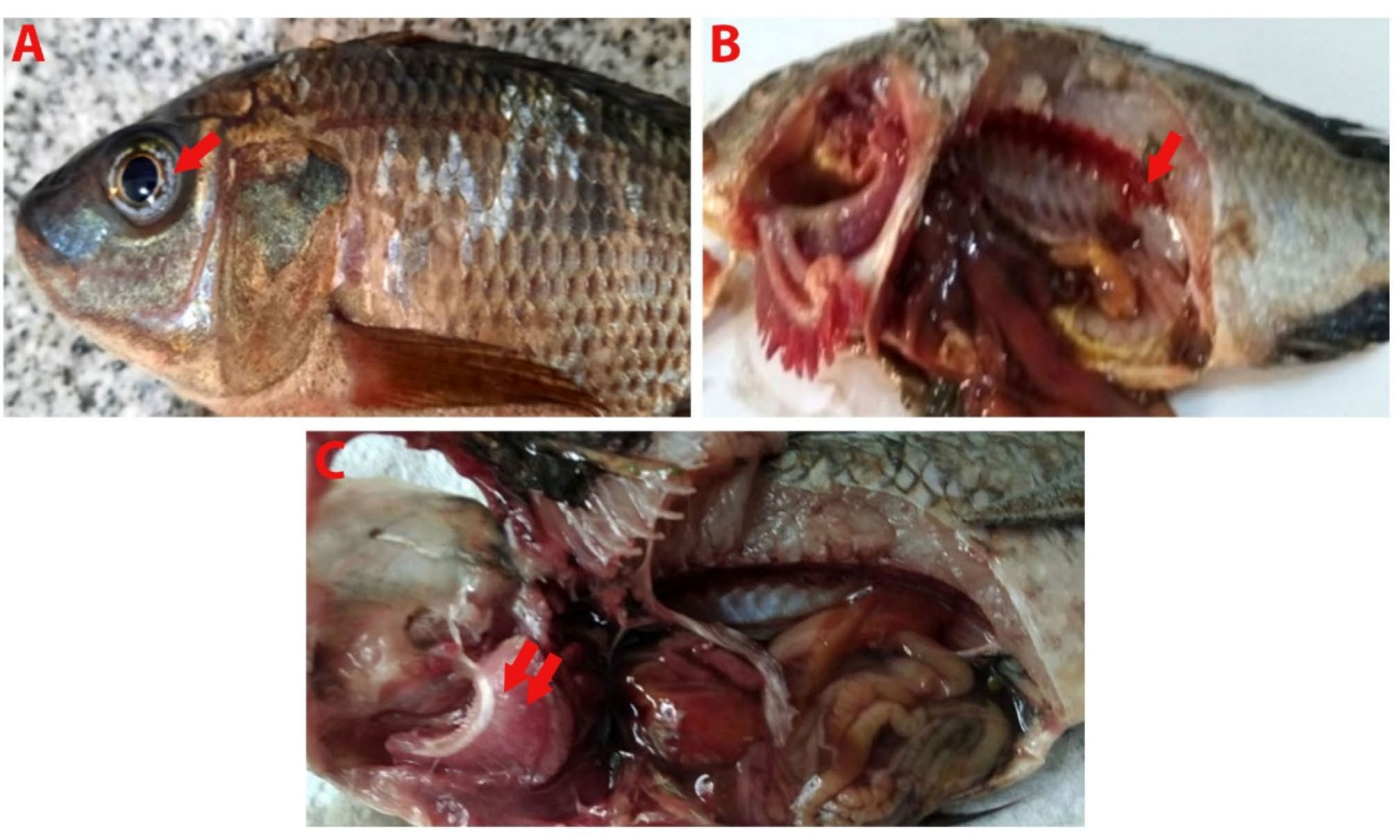
Nile tilapia infected with *Myxozoan spp.* showing multi white nodules in the eyes (inner wall of cornea) (arrow) (**A**), in the kidneys (**B**) and gill filaments (**C**)

**Fig. 3:**
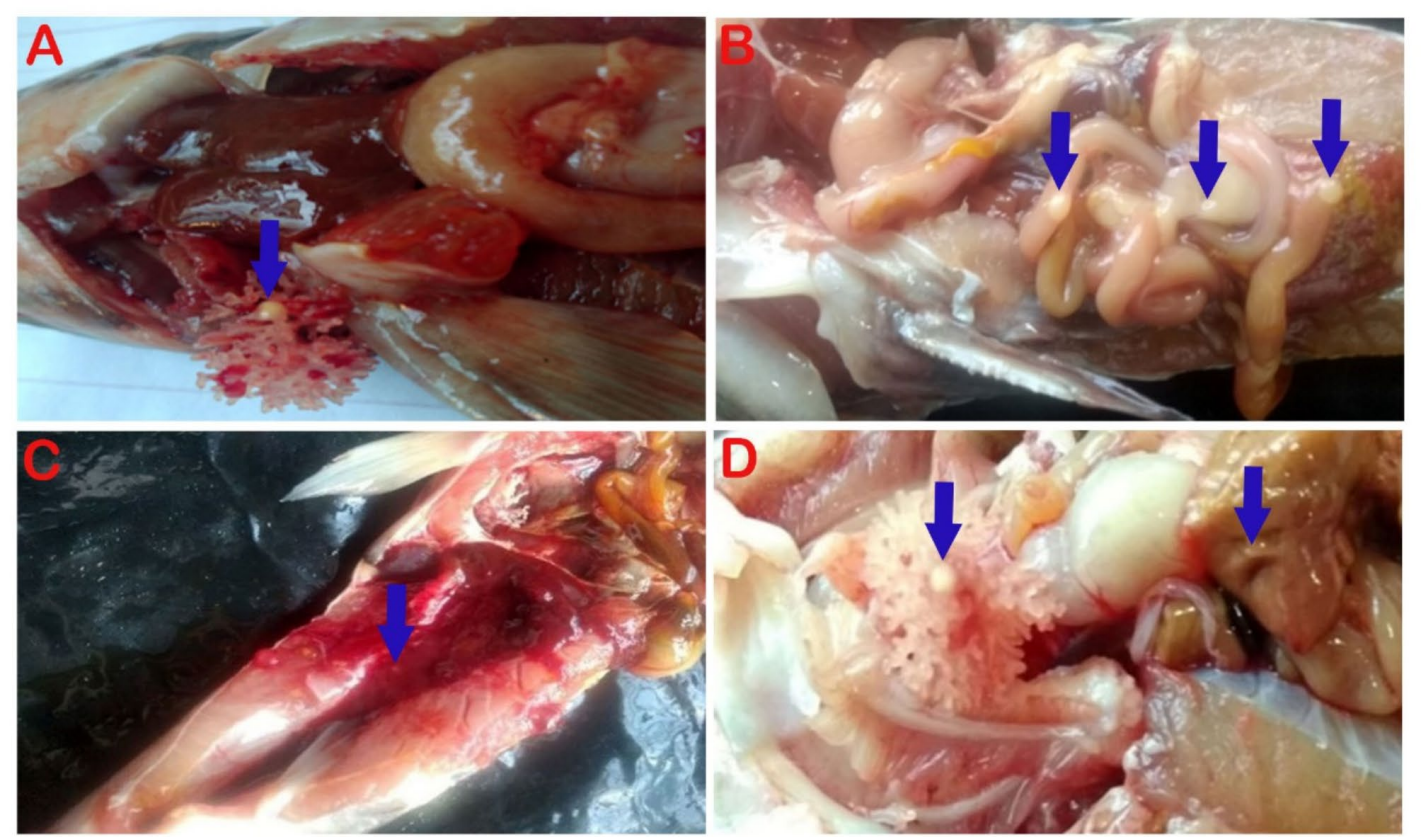
African catfish infected with *Myxozoan spp.* showed yellowish white nodules, in the dendritic organs (arrow) (**A**), intestine (arrows) (**B**), kidneys (arrow) (**C**). dendritic organ ( arrow) and in liver (arrow) (**D**)

Nile tilapia infected with *Myxozoan spp.* showed slight abdominal distension, uni- and bilateral exophthalmia and white nodules in the eye (inner wall of cornea) (Fig. 2A), posterior part of the kidneys (Fig. 2B), and gills (Fig. 2C). In African catfish, yellowish-white nodules were observed in the dendritic organs, intestine, kidneys, and liver (Fig. 3A-D).

### Microscopical observation

**Fig. 4:**
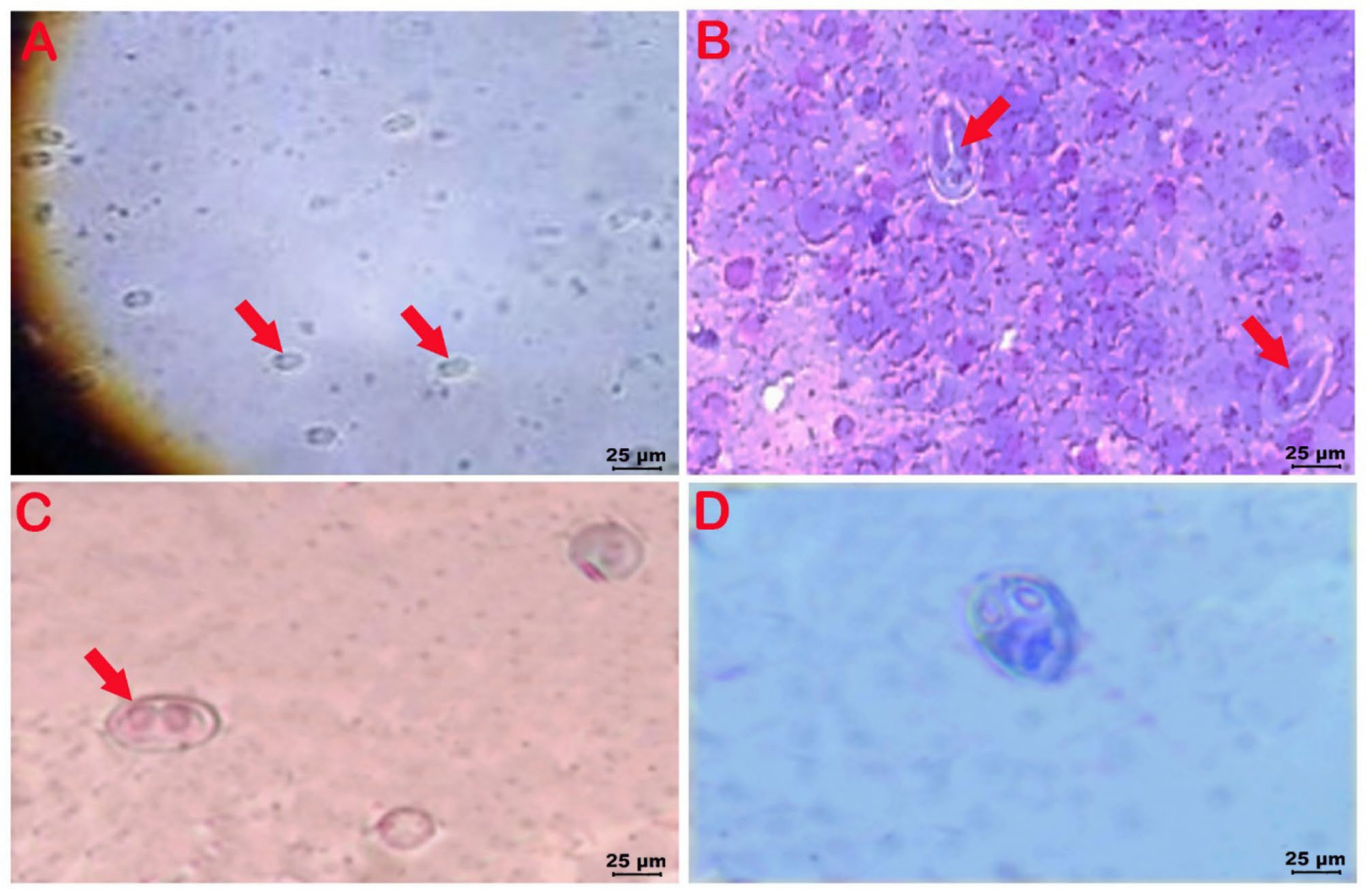
(**A**) Wet-mount preparation from nodules found in the kidneys of Nile tilapia showing *M. heterosporous* spores (x40); (**B**) Giemsa-stained smear from nodules recovered in the kidneys of Nile tilapia showing *M. amieti* spore (x40); (**C**) Giemsa-stained smear from nodules recovered in the eyes showing *M. brachysporus* spore (x40); (**D**) Giemsa-stained smear from nodules recorded in the kidneys showing *M. tilapiae* spore (x40)

*Myxosporean* spores isolated from different organs of Nile tilapia are presented in Fig. 4. *Myxobolus heterosporous* spores from the kidneys were ovoid, measuring (14.84–14.05 μm) × (9.65–10.11 μm). The two polar capsules were ovoid unequal in size occupying one-third of the spore and measuring (3.71–4.68 μm) × (3.04–4.12 μm) (Fig. 4A). *Myxobolus amieti* spores recorded from the kidneys were pyriform with pointed anterior end and rounded posterior end, ranging (10.19–12.64 μm) in length and (7.48–7.87 μm) in width. Their two polar capsules were elongated, and equal in size occupying more than half the length of spores measuring (6.83 ± 0.3 μm) in length and (2.92 ± 0.4 μm) in width (Fig. 4B).

*Myxobolus brachysporus* spores isolated from the eye and gills of Nile tilapia and kidneys of African catfish were ellipsoidal and well known for their width (42. 0 μm) exceeded than length (28.42 μm) with ovoid polar capsules measuring (9.84 ± 1.06 × 7.48 ± 0.35) µm (Fig. 4C). *Myxobolus tilapiae* spore recorded from the kidneys was relatively large ovoid (21.34 × 16.11 μm), with rounded anterior end and nearly wide posterior end Its polar capsules were ovoid, equal in size (6.33 ± 0.24 × 5.29 ± 0.36) µm, and sporoplasm occupying almost the spore size (Fig. 4D).

**Fig. 5:**
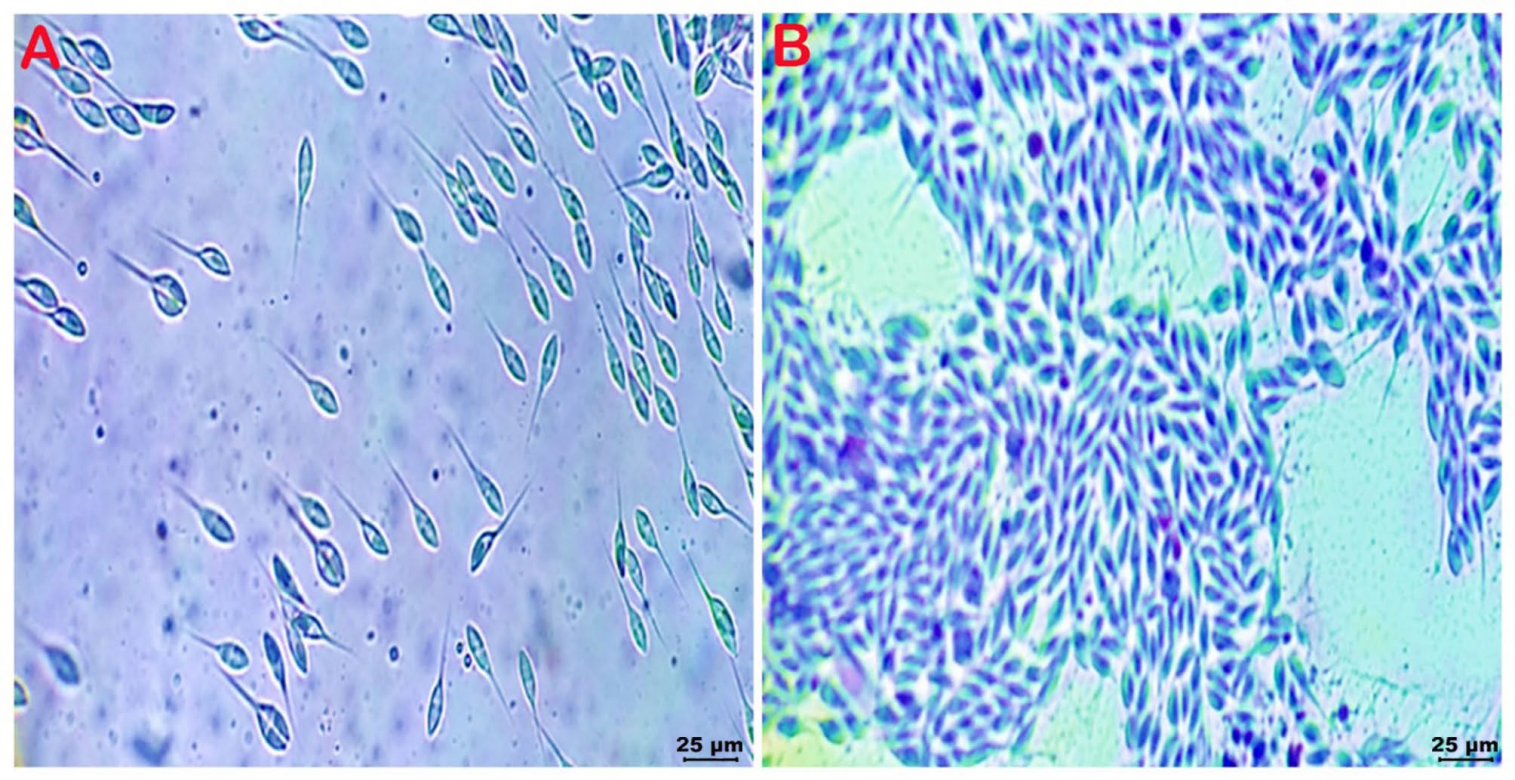
**A**: Wet-mount preparation from nodules recorded in the dendritic organ of African catfish showing *Henneguya spp.* spores resembles spermatozoon (X40), **B**: Giemsa-stained smear from nodules in the intestine of African catfish showing *Henneguya spp.* spores (X40)

In African catfish, the examined nodules from dendritic organs, kidneys, and intestines showed mature spores of *Henneguya spp*. which resemble spermatozoon. The spores were elongated anteriorly, had rounded anterior ends, were fusiform and had clear vacuoles (Fig. 5A.**B**). Moreover, *M. brachysporus* was also isolated from the kidneys of African catfish, similar to that recorded in Nile tilapia.

### Total and seasonal prevalence rate

The total and seasonal prevalence of *Myxozoan* infections among the examined Nile tilapia are presented in Table 3. *Myxozoan* infection in Nile tilapia revealed total prevalence 12% (48/400), representing 8.75% infection with *M. brachysporus* 2.75% infection with *M. amieti*, 0.25% infection with *M. heterosporous*, and 0.25% infection with *M. tilapiae.* The highest seasonal prevalence was 21% in spring, including (15% *M. brachysporus*, 5% *M. amieti*, and 1% *M. tilapiae).* In winter, the infection rate reaches 13%, including infection with *M. brachysporus and M. amieti* at the rate of 9% and 4%, respectively. Meanwhile, the seasonal prevalence in autumn was 9%, involving (7% *M. brachysporus*, 1% *M. amieti*, and 1% *M. heterosporous*). The lowest prevalence was 5% in summer, including infection with *M. brachysporus* at a rate of 4% and *M. amieti* at prevalence rate of 1% (Table 3. The total tissue susceptibility to *Myxozoan spp.* infection in Nile tilapia was as follows: from the eyes (*M. brachysporus and*, *M. amieti)* at a rate of 11%, from the kidneys (*M. amieti*,*M. heterosporous and M. tilapiae)* at a rate of 0.75%, and from gill filaments (*M. brachysporus)* at a rate of 0.25%.

**Table 3:** Total and seasonal prevalence of *myxozoan* infection in Nile tilapia

Seasons	No of examined fish	*Myxozoan spp*				No of infected fish	Infected %
		*M.brachysporus*	*M.amieti*	*M.heterosporous*	*M.tilapiae*		
Winter	100	9	4	0	0	13	13
Spring	100	15	5	0	1	21	21
Summer	100	4	1	0	0	5	5
Autumn	100	7	1	1	0	9	9
Total	400	35	11	1	1	48	12

In African catfish, total prevalence of *Henneguya spp.* infection was 24%, with seasonal prevalences 32%, 22%, 24 and 18% in winter, spring, summer, and autumn, respectively. *M. brachysporus was* recorded at a total prevalence of 2.5% and the seasonal infection rate was high in winter and autumn (4%), followed by spring (2%), with no record in winter (0%) (Table 4).

**Table 4:** Total and seasonal prevalence of *myxozoan* infection in African catfish

Seasons	No of examined fish	*M. brachysporus*		*H. suprabranchiae*		No of infected fish	Total Infected%
		No	%	No	%		
Winter	50	2	4	16	32	18	36
Spring	50	1	2	11	22	12	24
Summer	50	0	0	12	24	12	24
Autumn	50	2	4	9	18	11	22
Total	200	5	2.5%	48	24	53	26.5

### Molecular and phylogenetic tree building

**Fig. 6:**
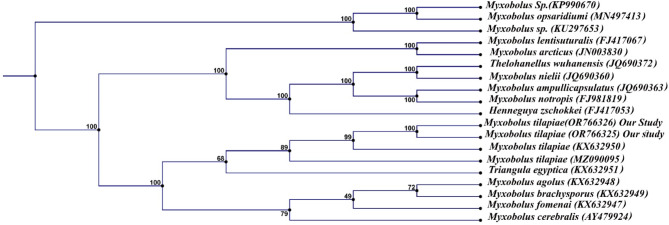
Phylogenetic tree was re-constructed based on the 18S rDNA sequences of *M. tilapiae* (OR766325 and OR766326) and its closest *Myxobolus species*. and *Henneguya spp*. using the neighbor-joining method

**Fig. 7:**
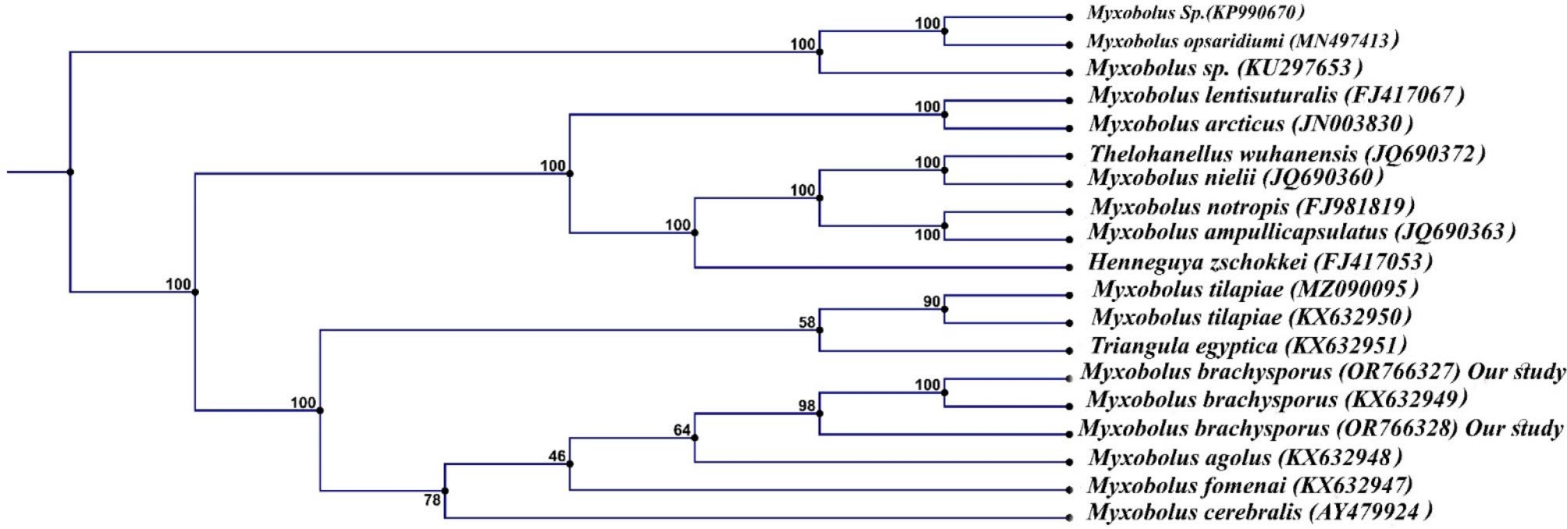
Phylogenetic tree was re-constructed based on the 18S rDNA sequences of *M. brachypterous (*OR766327 - OR766328) and its closest *Myxobolus spp.* and Henneguya species using the neighbor-joining method

**Fig. 8:**
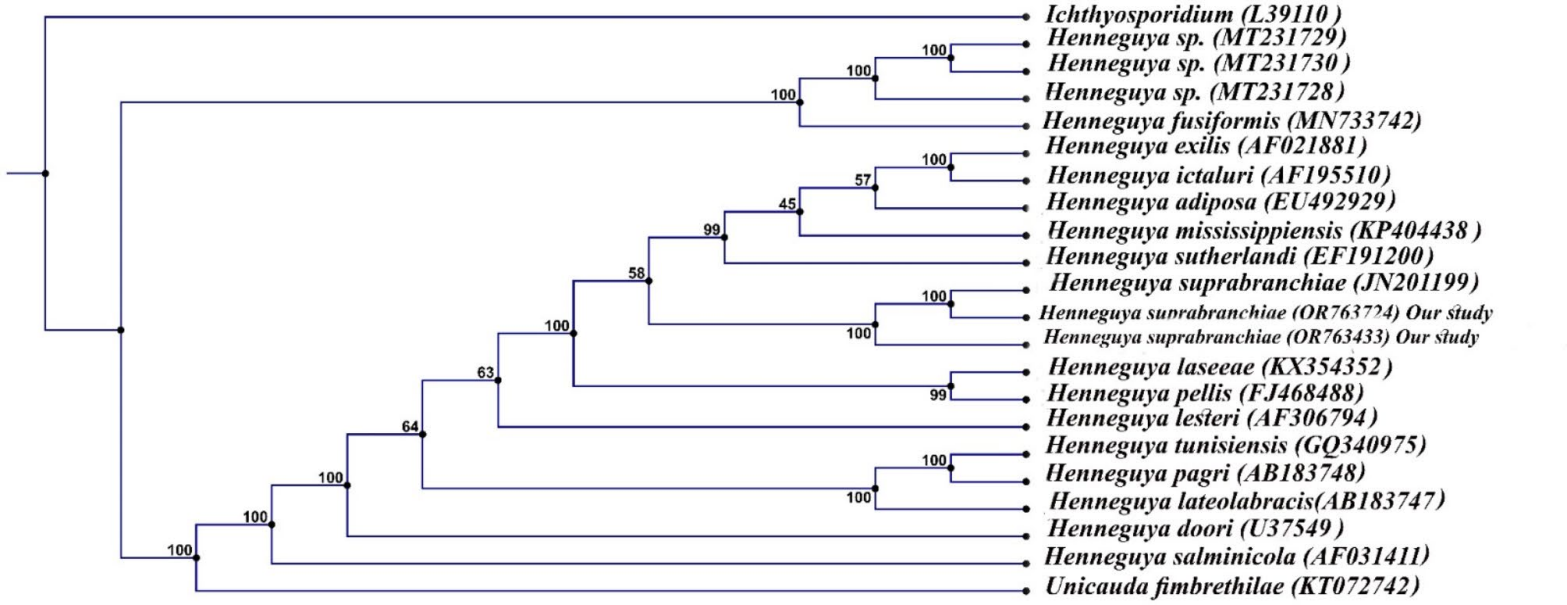
Phylogenetic tree was re-constructed based on the 18S rDNA sequences of *Henneguya suprabranchiae* (OR763724 and OR763433) and its closest *Henneguya* species using the neighbor-joining method

PCR amplification of the 18S rDNA for *Myxozoan spp.* isolated from Nile tilapia revealed 1300 bp and that isolated from African catfish showed 1000 bp. Phylogenetic analysis recorded *M. tilapiae* firmly embedded within the family Myxobolidae and showed 99% similarity to that of *M. tilapiae* (KX632950) and 89% similarity to that of *M. tilapiae* (MZ090095). Also, revealed 72% similarity with *M. brachysporus* (KX632949) and 68% similarity with that of *Triangula egyptica* (KX632951) (Fig. 6.*M. tilapiae* identified from Nile tilapia was deposited in GenBank under accession numbers (OR766325, OR766326). *M. brachysporus* sequences isolated from African catfish were deposited in the GenBank under accession numbers (OR766327, OR766328). It showed 100% similarity to that of *M. brachysporus* (KX632949),64% similarity to that of *M. agolus* (KX632949), and 78% with *M. cerebralis* (AY479924) (Fig. 7). The assembled sequence of 18S rDNA of *Myxozoan spp.* isolated from African catfish showed *H. suprabranchiae* that deposited in the GenBank under the accession number (OR763724, OR763433). *H. suprabranchiae* of this study showed 100% similarity to that of *H. suprabranchiae* described from *Clarias gariepinus* (JN201199) (Fig. 8).

## Discussion

Freshwater fishes are susceptible to parasitic diseases, including Myxosporean infection. In the present study, Nile tilapia infected with *Myxozoan spp.* showed slight abdominal distension, uni- or bilateral exophthalmia, and creamy white nodules of variable size around the iris, liver, gills, and kidneys. Similarly, macroscopic creamy whitish nodules in the eyes and gills of wild Nile tilapia infected with *myxobolus spp.* were recorded [12, 14, 45]. In addition, numerous white cysts of *Myxobolus dermatobius* have been recorded in the eyes of cultured Nile tilapia in Sharkia Governorate, Egypt [15]. In the present study, the clinical and postmortem lesions of henneguyosis, appeared as oval to round yellowish to white nodules in the dendritic organs, intestine, and kidneys of African catfish. Similar signs and lesions have been previously recorded [12, 17, 46–48].

Microscopic examination in this study revealed different *Myxozoan spp.* in different tissues. *M. brachysporus* spores isolated from the eyes and gills were ellipsoidal (42. 0 μm width, 28.42 μm in length), with ovoid polar capsules (9.84 ± 1.06 × 7.48 ± 0.35 μm). This species has been recorded in Nile tilapia in the kidneys, liver, and spleen [14, 18, 42, 49, 50]. *M. tilapiae* spores were also recorded in our study from kidneys which appear large ovoid with rounded anterior end and nearly wide posterior end measuring (21.34 × 16.11 μm). Mohammed et al. [13] recorded *M. tilapiae* from the gills of *Tilapia zilli* collected from the Nile River in Qena Governorate, Egypt. Abdel-Baki et al. [19] and Abdel- Gaber et al. [42] reported *M. tilapiae* from the kidneys measuring (15.3 ± 0.2 μm and 15.0 ± 0.5 μm) in length and (10.3 ± 0.1 μm and 11 ± 0.3 μm) in width, respectively. Moreover, *M. tilapiae* spores of an average (12.5 ± 0.67 μm in length × (5.7-± 1.2 μm in width) were observed in the gills of cultured Nile tilapia [20].

Moreover, in our study, *M. heterosporous* spores from the kidneys measuring (14.84–14.05) × (9.65–10.11) µm were recorded. Baker [51] recorded *M. heterosporous* spores (13 − 16 × 7–9.2 μm) from the gills of Nile tilapia, East Africa. Soror et al. [14] revealed *M. heterosporous* spores (18.52 μm length×11.54 μm width) from kidneys of Nile tilapia collected from El-Riah El-Tawfiki, Qaliobia Governorate, Egypt, and Eissa et al. [16] isolated the spore from the cornea of Nile tilapia at Abbasa Fish Farm, Sharkiya Governorate, Egypt. *M. heterosporous* spores (9.7 μm in length× 7 μm in width) were also recorded in the liver and intestines of naturally collected Nile tilapia from the Nile River [50]. In the current study the recorded spores of *M. amieti* from the kidneys appeared pyriform ranging from (10.19–12.64) µm in length and (7.48–7.87) µm in width with two equal elongated polar capsules (6.83 ± 0.3 × 2.92 ± 0.4 μm) occupying more than half the length of spore. *M. amieti* spores were previously recorded in the kidneys of Nile tilapia measuring (16.09 × 9.42 μm) with elongated polar capsule (9.91 × 3.09 μm) [14]. Our results were similar to the morphological description of mature spores of *Henneguya spp.* recorded in the organs of African catfish [12, 17, 31, 48, 52].

The overall prevalence of *Myxozoan* infections among the examined Nile tilapia in the current study was 12%, with the highest infection rate of 12% in spring and the lowest rate of 5% in summer. Shehab El-Din [45] recorded the total myxozoan infection of 10.6% in wild Nile tilapia with a seasonal prevalence of 3.03%, 2.08%, 0%, and 5% in winter, spring, summer, and autumn, respectively. Our results have been found to be lower than the data published by Soror et al. [14] who recorded 83.46% prevalence of *Myxosporean* infections in Nile tilapia, with the highest infection rate in autumn (95.08%) and the lowest rate was in summer (76%). In addition, Matter et al. [12] recorded that total prevalence of myxosporidiosis in Nile tilapia was 24.0%, and the highest seasonal prevalence was observed in winter (43.4%), whereas the lowest rate was recorded in summer (8%). Abdel-Baki et al. [19] and El-Asely et al. [37] respectively also reported higher infection rates with 61% and 100%. This difference could be attributed to differences in localities and water sources. Ali [53] reported that the highest prevalence of myxosporidiosis was in spring, explaining that parasites begin to form nodules in winter, the maximum number reaches in spring, and then starts to decrease after rupture of cysts to release spores in the environment.

In the present study, the infection with *M. brachysporus* reached 8.75% in Nile tilapia, and 2.5% in African catfish. On the other hand, Abdel-Baki et al. [18] recorded total infection rate of *M. brachysporus* in Nile tilapia collected from the Nile River was 51.9% and Georges et al. [49] observed 12.29% infection rate in Nile tilapia from Adamawa-Cameroon. *M. brachysporus* at prevalence of (12%) with the highest incidence rate of 14% in winter and 10% in summer was recorded by Abdel-Gaber et al. [42]. The examined Nile tilapia in the present study revealed total prevalence 0.25% for *M. heterosporous.* This result is lower than that reported by Georges et al. [49].

For *M. tilapiae* recorded in the present study, the total infection rates reached 0.25%, which only isolated in spring season. In contrast, Abdel-Gaber et al. [42] recorded that *M. tilapiae* among the examined Nile tilapia exhibited overall prevalence of 6%, representing 8% incidence in winter and 4% occurrence in summer. In addition, Georges et al. [49] observed a 15.14% infection rate with *M. tilapiae.* These results could be attributed to the availability of intermediate hosts and the increase in fish feeding activity at warm temperatures [54, 55]. Moran et al. [56] attributed the variation between the seasonal prevalence of myxosporean infection, which may be due to variation in the environmental conditions, and the time of exposure which may extended to 5–6 month.

The total prevalence of *henneguyasis* was 24%, with seasonal prevalence of 32%, 22%, 24%, and 20% in winter, spring, summer, and autumn, respectively. The overall prevalence of infection with *Henneguya spp.* which have been previously reported was 18% [57], 40% [58], and 43.65% [12]. Furthermore, *Henneguya spp*. in Nile tilapia was recorded by Ramadan et al. [59] at a rate of 8.4% [12]. The difference in infection rates could be attributed to fish feeding behavior as a carnivorous species that assists in the transmission of more enteric parasites by feeding on aquatic animals that harbor the infective stage of these parasites or even fed on small infected fish [23].

The identification of diverse species of *myxosporidian* spores on a morphological and morphometric basis, is more difficult due to their abundance. Therefore, molecular assays based on small subunit ribosomal DNA gene sequences are the most sensitive method for accurate identification [60]. Molecular identification using 18s rDNA gene sequences of *Myxozoan spp.* in the present study, recorded *M. tilapiae* from the kidneys of Nile tilapia which deposited in the GenBank (OR766325, OR766326) and showed 89 − 99% similarity to that of *M. tilapiae*,72% similarity with *M. brachysporus*, and 68% similarity to that of *Triangula egyptica.* Eissa et al. [20] recorded that *M. tilapiae* (MZ090095) showed 99.46% identity with *M. tilapiae* (KX632950), 98.85% similarity with *M. brachysporus*, 98.77% similarity with *Triangula egyptica*, and 97.45% similarity with *M. cerebralis*. Abdel-Gaber et al. [42] reported that the amplified and sequenced SSU rDNA gene regions for the recovered *Myxospora sp.* from Nile tilapia had 95% identity with all *Myxobolus* species available in GenBank. In this study, *M. brachysporus* that isolated from the kidneys of African catfish with accession numbers (OR766327, OR766328) showed 100% similarity to that of *M. brachysporus* (KX632949),64% similarity to that of *M. agolus* (KX632949), and 78% identity with *M. cerebralis* (AY479924) in Nile tilapia in Egypt. In the current study, molecular assays of *Henneguya spp.* recovered from the dendritic organs and kidneys of African catfish recorded *H. suprabranchiae* sequences deposited in GenBank (OR763724, OR763433), which exhibited 100% similarity to that of *H. suprabranchiae* (JN201199) reported by Morsy et al. [31].

## Conclusion

The results of the present study revealed that Nile tilapia and African catfish collected from natural water resources were infected with *myxozoan spp*. The prevalence was higher in African catfish than that in Nile tilapia. Molecular and phylogenetic tree building as an important diagnostic tool, confirmed *M. tilapiae* in Nile tilapia, and *M. brachysporus* and *H. suprabranchiae* in African catfish. Further investigations are needed to estimate the economic impacts of myxozoan infection on natural captures and total productivity.

## Electronic supplementary material

Below is the link to the electronic supplementary material.

Supplementary Material 1

Supplementary Material 2

Supplementary Material 3

## Data Availability

https://www.ncbi.nlm.nih.gov/nuccore/OR766325 https://www.ncbi.nlm.nih.gov/nuccore/OR766326 https://www.ncbi.nlm.nih.gov/nuccore/OR766327 https://www.ncbi.nlm.nih.gov/nuccore/OR766328 https://www.ncbi.nlm.nih.gov/nuccore/OR763724 https://www.ncbi.nlm.nih.gov/nuccore/OR763433.
