# Supplementary figures and images for: Clinical signs, morphological and phylogenetic characterization of Myxozoan spp. infecting Nile tilapia, Oreochromis niloticus and African catfish, Clarias gariepinus in Qalyubia Governorate, Egypt
Source: BMC Vet Res. 2024 Nov 27;20:530. doi: 10.1186/s12917-024-04378-0 (PMC11600721; doi:10.1186/s12917-024-04378-0)

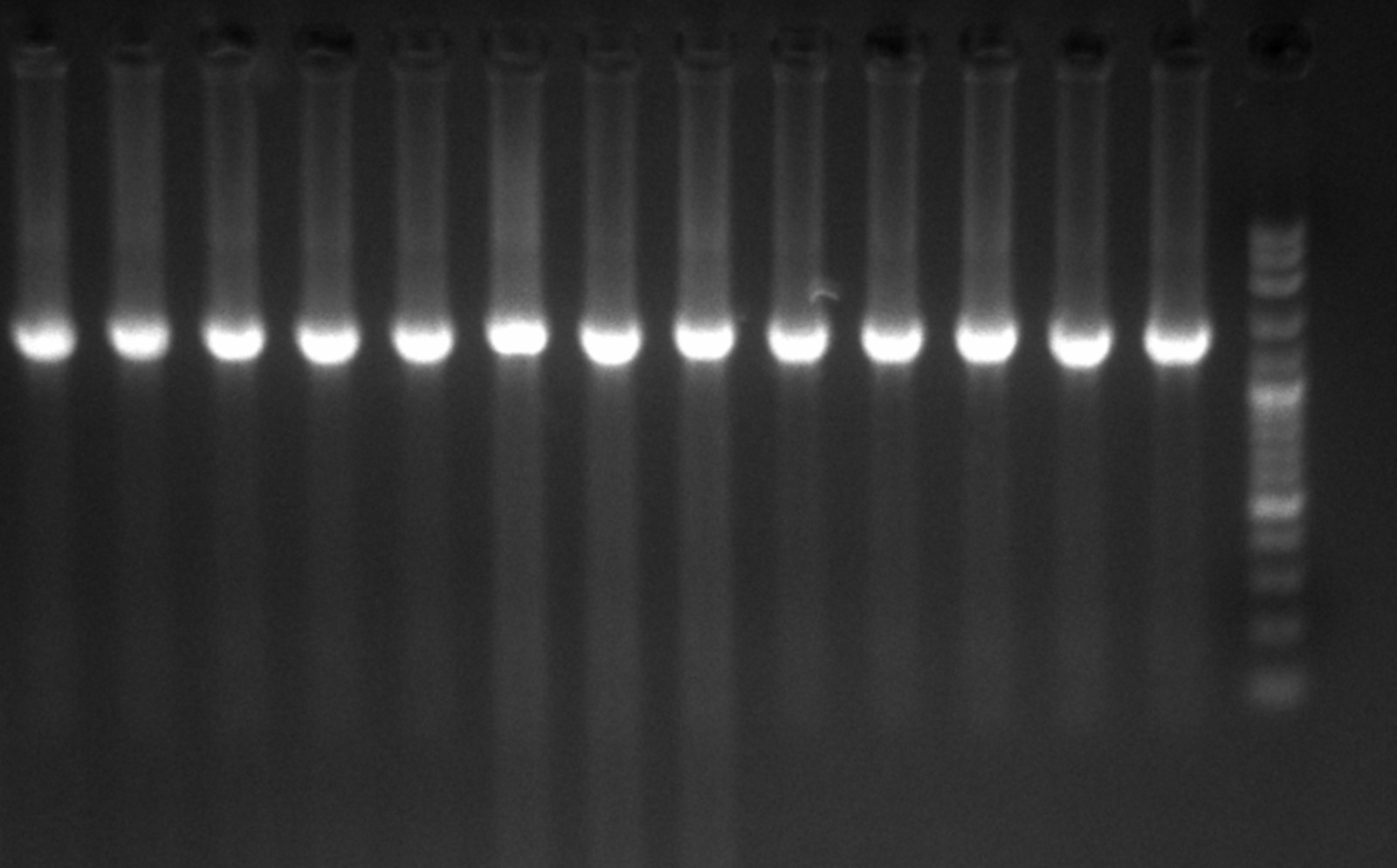


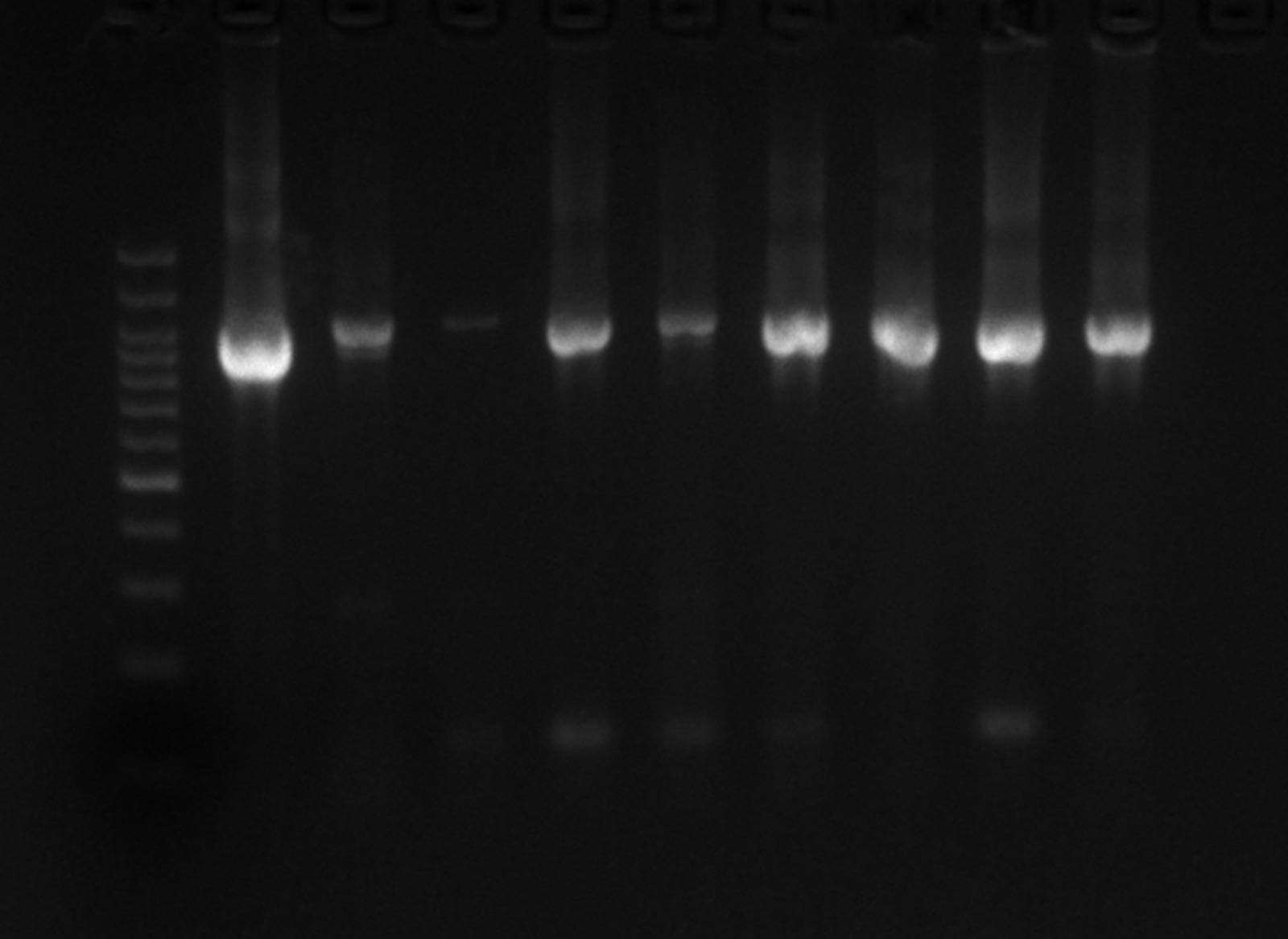

Supplement: Supplementary file 1 — Supplementary Material 1 [file 12917_2024_4378_MOESM1_ESM.docx]

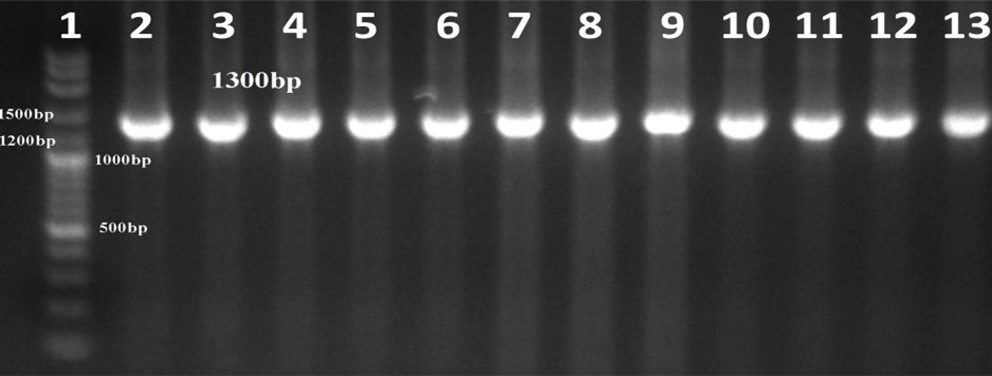

Supplement: Supplementary file 2 — Supplementary Material 2 [file 12917_2024_4378_MOESM2_ESM.jpg]

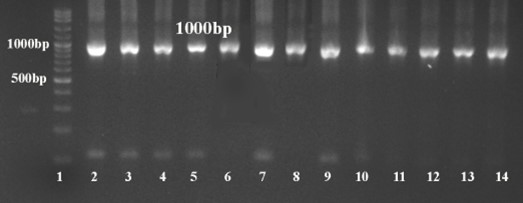

Supplement: Supplementary file 3 — Supplementary Material 3 [file 12917_2024_4378_MOESM3_ESM.jpg]
